# Reducing Moral Stress in Veterinary Teams? Evaluating the Use of Ethical Discussion Groups in Charity Veterinary Hospitals

**DOI:** 10.3390/ani13101662

**Published:** 2023-05-17

**Authors:** Vanessa Ashall

**Affiliations:** Science and Technology Studies Unit (SATSU), Department of Sociology, Heslington East Campus, University of York, York YO10 5GD, UK; vanessa.ashall@york.ac.uk

**Keywords:** moral stress, mental health, veterinary, ethical dilemma, euthanasia, animal, practical barrier, relational barrier, ethical discussion

## Abstract

**Simple Summary:**

This study aims to evaluate the use of ethical discussion groups for reducing moral stress in veterinary teams. The data analysis is based on focus groups and individual interviews with veterinary team members working in charity veterinary practice. Moral stress is described as an everyday experience in the lives of veterinary team members and is caused by uncertainty about their ability to fulfill their ethical obligations. Moral stress is shown to be cumulative and can interact with other forms of stress. Distinct practical and relational barriers to ethical action are identified and proposed as contributors to moral stress, whilst the potential impact of moral stress on team members’ quality of life and mental health is highlighted. Results show that regular facilitated ethical group discussions may reduce moral stress in the hospital setting, particularly through familiarization with others’ roles and perspectives and supporting one another’s ethical decision-making. The article concludes that moral stress is an important and poorly understood problem in veterinary practice and that further development of regular facilitated ethical group discussion may be of considerable benefit to team members.

**Abstract:**

This study examines experiences of veterinary moral stress in charity veterinary practice and qualitatively evaluates the role of ethical discussion in reducing veterinary moral stress. Results are drawn from a thematic data analysis of 9 focus groups and 15 individual interviews with veterinary team members from 3 UK charity veterinary hospitals. Moral stress is described as an everyday experience by participants and is caused by uncertainty about their ability to fulfill their ethical obligations. Moral stress is shown to be cumulative and can interact with other forms of stress. Distinct practical and relational barriers to ethical action are identified and proposed as contributors to moral stress, and different team members experience different barriers within their roles. The potential impact of moral stress on team members’ quality of life and mental health is highlighted. Results show that regular facilitated ethical group discussions may reduce moral stress in the hospital setting, particularly through familiarization with others’ roles and ethical perspectives and through supporting one another’s ethical decision-making. The article concludes that moral stress is an important and poorly understood problem in veterinary practice and that further development of regular facilitated ethical group discussion may be of considerable benefit to team members.

## 1. Introduction

The mental health of the veterinary profession has become an area of increasing concern. Recent media articles discuss a profession at ‘breaking point’, reporting a mass exodus of vets from the profession in the context of the COVID-19 pandemic, a pet ownership boom, exhaustion, and burnout [1]. In the UK, there has been an increasingly visible focus on veterinary mental health within the profession with the rollout of dedicated research funding [2], tailored mental health training [3], alongside ongoing professional crisis support [4]. The potential impact of poor mental health within the profession is recognized as severe, with veterinary suicide rates potentially three times higher than the national average [5]. The causes of poor mental health in veterinary professionals are recognized as multifactorial, with occupational, personal, and individual factors all likely to be implicated [6]. Moral stress was highlighted as an issue for veterinarians performing euthanasia of shelter, unwanted, or “surplus” animals in the 1980s [7]. The specific relationship between ethical dilemmas in veterinary practice and workplace stress was further explored by Bachelor and McKeegan (2012) [8], who used survey work to show that vets regularly face ethical dilemmas and that they find these stressful. The idea that workers may suffer negative psychological consequences through exposure to moral challenges, as distinct from other causes of workplace stress and burnout, is also well recognized within both the medical and other professions [9].

This paper is focused on the concept of moral stress and its relationship with veterinary ethical decision-making as one potentially important cause of poor mental health in veterinary teams [8]. The phrase “moral stress” has been well-defined outside the profession; for example, “*moral stress is a psychological state born of an individual’s uncertainty about his or her ability to fulfill relevant moral obligations*” [10]. Specific use of this phrase within the veterinary literature is more recent, however, with contemporary work describing moral stress as “*The feeling of not being able to do what you believe to be ‘the right thing’ because of constraining personal, professional, organizational or client factors*” [11] (p. 421). Veterinary moral stress is distinguished from veterinary moral distress by Gibson and Quain (2022) [11], who suggest that moral distress occurs when one transgresses one’s deeply held personal beliefs [12].

Moral *distress* is also explored by Moses et al. (2018) [13], who use survey data to show that for 78% of respondents, not being able to do the right thing for a patient caused veterinary staff moderate to severe distress. Moral distress is encountered frequently in veterinary practice, Moses et al. (2018) report that a majority of respondents feel conflicted over what care it is appropriate to provide [13]. Furthermore, 70% of respondents felt that the obstacles they faced that prevented them from providing appropriate care caused them or their staff moderate to severe distress. The authors suggest that ethical conflict and resulting moral distress may be an important source of stress and poor well-being that is not widely recognized or well-defined and that “*even less has been written about how veterinarians feel about and cope with these kinds of situations*” [13] (p. 2115). In addition, Moses et al. (2018) suggest that whilst veterinary surgeons frequently encounter ethical conflicts, which cause them moral distress, they rarely recognize these situations as ethical or moral in nature. A key conclusion from this work is that “*recognizing, acknowledging, and labeling conflict and distress as ethical in nature are important first steps in combating moral distress*” [13] (p. 2121).

Williamson et al., 2022 [14], have recently described moral *injury* in the UK veterinary profession, which they describe as the profound psychological distress experienced following an event involving acts of perpetrating, bearing witness to, or betrayal by trusted others which violate one’s deeply held moral beliefs or expectations [15]. Williamson et al., 2022, demonstrate a connection between experiences of potentially morally injurious events (PMIEs) and adverse mental health outcomes in UK veterinary practitioners. With regards to the role of ethical discussion in alleviating such experiences, the authors suggest that “*interventions which foster open discussions, and reflections on, PMIEs, as well as training to prepare VPs for circumstances where they may not be able to provide ‘Gold Standard’ care, may be protective”* [14] (p. 6).

Whilst the specific relationship between the terms moral stress, moral distress, and moral injury has not been made explicit in the veterinary literature, Litz and Kerig (2019) describe a “*continuum of morally relevant life experiences and corresponding responses, with varying magnitudes and impacts*” [16] (p. 344). The authors describe moral challenges, stressors, and injurious events as occurring with reducing frequency and increasing negative impact. They suggest that moral frustration, moral distress, or moral injury may be experienced as a result of these varying moral experiences. As such, this current study tests the theory that moral stress is an everyday concept, which relates to Litz and Kerig’s 2019 definition of moral frustration [16] and represents a more frequent but less severe example of the psychological harms caused by ethical challenge in the veterinary clinic, which has been previously described as moral distress [13,17] and moral injury [14].

The role of ethical group discussion as a management tool for veterinary moral stress is explored in this paper with specific reference to a recent publication by the veterinary charity the People’s Dispensary for Sick Animals (PDSA), which described the charity’s work to advance animal welfare and ethics in its hospitals. As part of that work, staff surveys demonstrated that less than half (49%) of PDSA hospitals are completely satisfied with the level of discussion about ethically challenging cases [18]. In response, the organization has proposed the development of open discussion centered on ethical reasoning, ethical dilemmas, and reflection on cases as a possible management tool in reducing conflict and moral stress within their teams:

*“Different members of a clinical team can draw different legitimate conclusions on what is an ethical way to proceed with a given clinical case; such differences can be a source of conflict and moral stress if they are not understood and respected. Open discussion on ethical reasoning and ethical dilemmas can help ensure decisions are arrived at and supported by as many team members who will be involved with a patient’s care (or affected by a patient’s euthanasia) as possible while open reflection on recent cases and decisions can inform team approaches to future similar cases.”*

[18] (p. 7)

Wensley et al. (2020) [18] proposed that ethical discussion may reduce conflict and moral stress within their teams, building on longstanding calls to develop forums for ethical discussion within veterinary practices [19,20]. Most recently, ethical discussion in veterinary practice has been developed through the survey work of Quain et al. (2022), who described the beneficial effects of “virtual ethics rounds” for veterinary professionals and their potential to mitigate moral distress [17]. Gibson and Quain (2022) also propose the development of face-to-face ethical discussions within the practice setting as one element within the development of a Clinical Ethics Support Service (CESS) in practice [11]. In this present study, a longitudinal qualitative evaluation of consecutive ethical group discussions based in charity veterinary hospitals develops Quain et al.’s (2022) survey analysis of standalone virtual ethics rounds, which involved staff across multiple veterinary and animal welfare organizations [11]. This present study’s embedding of ethical group discussion within a specific organization also responds to Millar’s (2018) call for institutional responsibility in advancing veterinary ethics [20].

The importance of structure and facilitation in ethical group discussion has also been previously identified, with the use of an external facilitator who is ideally ethically trained being promoted by Gibson and Quain (2022) [11]. Quain et al. (2022) describe a facilitated “virtual ethics round” involving an experienced veterinarian and ethicist, whereby participants were introduced to the concepts of moral stress, moral distress, and moral injury, followed by a discussion of different courses of action relating to ethically challenging situations, which the participants had encountered [17]. Their virtual ethics rounds discussion is described as incorporating relevant laws or codes of practice, professional responsibilities, and key ethical theories, and the participants were ultimately asked to choose and justify one course of action.

In this study, a different approach to structure and facilitation of ethical discussion was trialed, although an experienced clinician and ethicist was, again, responsible. This study’s approach responds to recent work proposing the use of the Veterinary Ethics Tool (VET) [21] for structuring ethical discussions of all relevant aspects of clinical veterinary ethics. The VET aims to provide a framework for methodical clinical ethical decision-making and may “*facilitate discussion among clinical staff, especially in multidisciplinary settings, where ethical decision-making often falls to the primary clinician with varying input from other members of the team*” [21] p. 6. Other ethical tools, such as the ethical matrix [22], have previously been proposed for use in the veterinary setting, but for this study, the VET was chosen for its relative simplicity and its focus on the multiple relationships at work in the veterinary clinic, which contribute to the relational complexity of veterinary ethical decision making. The VET’s exploration of the reasons why ethical decision-making may not always be solely based on the best interests of the patient provides a useful access point to conversations centered on moral stress as an inability to achieve ethical obligations in the veterinary clinic.

The aim of this work is to further develop understanding of veterinary moral stress and to qualitatively evaluate the use of regular facilitated ethical group discussions in charity veterinary hospitals as a route to reducing moral stress in veterinary teams.

## 2. Materials and Methods

This is a longitudinal qualitative empirical ethical study. The paper reports a thematic analysis of focus group and interview data concerned with moral stress and ethical discussion in charity veterinary hospitals. The analysis is used to evaluate the usefulness of ethical group discussion in charity veterinary practice and, specifically, its role in reducing moral stress. The data analysis is also used as the foundation to develop broader normative arguments concerning veterinary ethical relationships and responsibilities.

### 2.1. Study Design and Data Collection

This study is an example of co-designed research, described as the meaningful involvement of end-users in research [23]. Recently, there has been a growing interest in research co-design with the purpose of maximizing research acceptability, applicability, and impact and addressing longstanding issues around power and depth of involvement in research [24]. In this study, the researcher and the veterinary charity worked together with the aim of exploring the use of ethical group discussion within their hospitals. The study was designed to be practical and achievable for the organization’s staff; as such, both the researcher and the PDSA took an active role in the study design, with the researcher subsequently conducting the research and analyzing the data. An in-depth qualitative approach was chosen due to the specific skills of the researcher, the suitability of this approach for making sense of *meaning* in healthcare settings [25], and the desire to achieve more detailed and contextual information about an area that has recently been studied using survey methods [17].

The focus group discussions involved up to six team members who usually worked together in one hospital. Potential participants were identified by the employing organization and were selected purposively [26] to achieve maximum variation in group makeup, ideally including both veterinary surgeons and veterinary nurses, junior and senior staff, and, where possible, a non-clinical team member. Participants were given an information sheet containing details of the project and were invited to ask the researcher any questions before providing written informed consent if they wished to participate. The information sheet also included details of internal and external veterinary mental health support services, which participants could access if they chose, to discuss any of the issues raised during the focus group conversations.

The focus groups were held across three different hospitals, identified by the organization, at different geographical locations in the UK and representing differing hospital sizes and socioeconomic backdrops. This study is longitudinal, and focus group discussions were held with the same participants over three consecutive one-hour sessions with the aim of allowing the participants to become familiar with ethical discussion as a group and to reflect on their experiences over a period of time. To facilitate the focus groups, the participants were all removed from the rota for a period of one hour when convenient for the hospital; sessions were held weekly or at longer intervals, depending on the availability of team members. Whilst in-person focus groups were initially planned, ongoing concerns regarding COVID-19 and restrictions to visitors at the hospitals meant that Zoom was ultimately employed to connect the researcher and the participants remotely [27]. Wherever possible, however, the team members were all in the same room at the hospital during the focus group sessions. Individual interviews were offered to all participants after their three focus group sessions had been completed. The individual interviews took place over Zoom at a time that was mutually convenient for the participant and the researcher. The individual interviews aimed to give each participant the opportunity to speak to the researcher in confidence and to explore their personal experiences in more depth.

Focus groups and interviews were semi-structured [26], with a guide being used by the interviewer to focus the conversation on specific topics of interest, but as far as possible, the conversation was allowed to develop naturally, fitting the description of a “*conversation with a purpose*” [28] (p. 102). The same structural format was followed for each focus group and for the individual interviews. During the first part of the conversation, the researcher used PowerPoint slides to introduce the PDSA’s findings that over half of the hospitals were currently dissatisfied with the level of ethical discussion [18] and invited the participants’ comments; next, the concept of moral stress was introduced and a definition provided [10] before comments were again invited; finally, the Veterinary Ethics Tool [21] was shared (via PowerPoint and as paper copy), and comments on the use of this tool were invited. For the second half of the focus groups, participants were invited to share their experiences of specific cases where ethical decision-making was difficult or where they had experienced moral stress. At first, the researcher suggested the topics of end-of-life care and the COVID-19 pandemic to start conversations within the groups until participants felt confident introducing their own topics for discussion. At subsequent focus groups and individual interviews, the same format was followed, allowing participants to build on their previous reflections as they gained greater familiarity with the concept of moral stress and the VET and became more confident in discussing their own experiences. The researcher allowed conversations to develop naturally as far as possible [28] and used only the definition of moral stress and the VET to help participants structure their ethical reflections where this was considered helpful.

The researcher did not introduce any further information to participants by way of ethical theory or identifying alternative clinical approaches. As such, the focus group discussions differed from the previously reported “ethics rounds” [17] in that they were less structured and had the aim of recording in real time the detail of facilitated ethical discussion within the existing teams rather than providing any form of ethical training for participants. The researcher’s dual role as a facilitator and researcher is in line with conventional constructivist approaches to qualitative data collection, where the researcher’s active role in data generation must be acknowledged [29]. Here, the researcher’s specific training and expertise in veterinary ethics will inevitably have influenced their facilitation and their analysis of the data, although their primary aim in this project was not to provide guidance or education in veterinary ethics, ethical theory, or ethical reasoning to participants, beyond that encompassed within the definition of moral stress and the published ethics tool, which was provided. This research should also be located in the context of the researcher’s own position, whereby they have historic personal experience of veterinary ethical conflict and moral stress. The researcher identifies with the participants as veterinary colleagues and is open about their own professional identity. The researcher is trained and experienced in qualitative social scientific research and veterinary ethics, and their role in this project was as an independent academic researcher.

### 2.2. Data Handling and Data Analysis

Data from focus groups and individual interviews were recorded using the inbuilt facility provided by Zoom [27]. The voice recordings were encrypted and sent via secure file transfer to a transcriber who had signed a confidentiality agreement. Anonymized, decontextualized, and cleaned [30] transcripts were returned to the researcher via secure file transfer and stored securely on the University Server. Voice recordings were deleted once the anonymized transcripts were obtained.

Thematic analysis was applied to the data using the qualitative research management software N-Vivo Release 1.7 (QSR International). Codes were identified through repeat reading of the transcripts to identify recurrent words, meanings, or phrases [26]. Codes were grouped into clusters and, finally, collapsed into themes using both inductive and deductive methods [26]. As such, some unexpected themes, such as the identification and separation of practical and relational barriers to ethical action, were generated during the data analysis, whilst veterinary relationships and the emotions associated with moral stress were explored intentionally as “a priori” themes [31], following discussions of the VET [21] and the published definition of moral stress [10].

## 3. Results

The results are divided into two sections, which address the study’s aims to develop an understanding of moral stress in charity veterinary teams and to evaluate the teams’ experiences of ethical group discussion. Within each section, three analytic themes structure the presentation of the results, although these themes do overlap and relate to one another. In the first section, an exploration of veterinary moral stress is structured around the themes of definition, practical and relational barriers to ethical action, and the impact of moral stress. The second section on experiences of ethical discussion is structured around the themes of practical considerations, the value of ethical reflection, and reducing moral stress.

### 3.1. Veterinary Moral Stress: Definitions; Causes; and Impact

This section describes how the participants understood the concept of moral stress, the specific barriers to resolving ethical challenges which created moral stress within their teams, and the potential impact of moral stress on veterinary team members.

#### 3.1.1. Defining Moral Stress

The researcher provided participants with a definition of moral stress [10]. Whilst participants were largely unfamiliar with the term itself, they reported recognizing the specific feeling and situation:

*“I probably hadn’t heard it named moral stress or anything, but I definitely had felt it and seen it before. Interview 1.*

*Yeah, I mean, I think… I think you’d be hard-pressed to find someone who hadn’t. Even like just a normal person who wasn’t a vet. But, like yeah, I definitely, in a veterinary context, have experienced it.” Interview 4.*

The participants identified closely with the concept of moral stress as a perceived inability to act on relevant moral obligations:

*“You always want to feel that your values and your actions remain aligned. Once something’s out of whack, there’s gonna be some inner conflict wanting a different course of action or a different situation.” Focus Group 4.*

Team members reported finding the phrase moral stress useful and intuitive as a way to help them describe their experiences:

*“I think it kind of, it does what it says on the tin!” Interview 3.*

*“Because as soon as you say it, I kind of know exactly what situations can cause me moral stress!” Interview 2.*

Whilst specific ethical conflicts were discussed for their role in causing moral stress, the participants also reported a more insidious and everyday uncertainty surrounding their ability to fulfill their ethical obligations:

*“So, the actual kind of moral stress [was] of thinking about those animals day-in, day-out, finishing work at six o’clock, but staying on to help the nurse that was on a call with the patients because I wanted to make sure that everybody got the same level of care that I felt was needed.” Interview 1.*

Moral stress was described as something that can accumulate over several cases or periods of time, and that could also become incorporated with other forms of stress:

*“So you know, I think for me it’s something that can build up over time. So maybe it’s just not one animal, but it’s maybe one or two over a week or so, and then maybe something at home, and then it builds up for me.” Interview 2.*

The study results provide important detail on daily experiences of moral stress, alerting us to the possibility of cumulative moral stress, where responses to several cases interact with one another or with other forms of stress.

In summary, existing definitions of moral stress resonated with veterinary team members. Although they were not familiar with this term, they found it helpful to describe their own experiences. Moral stress was experienced daily to varying degrees and was understood to be an unavoidable part of veterinary practice.

#### 3.1.2. Practical and Relational Barriers to Ethical Action

In line with the proposed definition of moral stress as an inability to act ethically, the participants identified specific obstacles [8,13] that created this problem for their teams. Efforts to achieve ethical outcomes were frustrated by either practical obstructions, such as the need to fairly allocate limited funds or a lack of time, or by relationships with other individuals, such as colleagues and animal owners who had different opinions on what the correct course of action might be. A significant practical cause of moral stress was the presence of financial barriers to undertaking veterinary treatments in the interests of the animals. This was particularly difficult for team members when the animal was young or when the alternative outcome was euthanasia:

*“I feel it sometimes, even with our clients, with things we can’t do, so you know, a young dog with a [complex] fracture repair that’s not something we have the capability to do here, but you could send to a specialist and can’t do. I mean, that’s outside our scope, you know. […] So we [amputated] … it’s leg. And we’re decreasing that animal’s quality of life, but there’s nothing [else] we could do.” Focus Group 9.*

Participants identified other practical obstacles to their desired clinical outcomes, including organizational policy, such as the number of pets which could be treated, and necessary treatment protocols:

*“You have to almost be like … here’s the ethical decision that my boss has made, and here’s their justification for it, and like you have to present that almost as like de facto the right position. Even if you don’t agree. And that’s hard.” Interview 4.*

Team members also recognized obstacles in terms of their own time and workload on their ability to achieve desired outcomes:

*“I try my hardest to treat animals late in the day when you’re running behind the same as earlier in the day […], so I guess it’s just trying to do the right thing each time when you’re busy and running behind and tired after a long day.” Focus Group 8.*

Through narratives centered on practical barriers to ethical action, team members generally accepted that they had no alternative but to act according to the identified practical options, even when this caused them moral stress.

An additional cause of moral stress was the impact of human relationships on the achievement of desired clinical outcomes, described here as relational barriers. Tensions between team members and animal owners were frequently encountered in daily practice; the analysis illustrates that different relational barriers to ethical outcomes were experienced by veterinary surgeons, veterinary nurses, and non-clinical team members:

*“So the moral stress that a nurse is going to feel is because they think they can’t get a vet to do what they think is needed. Whereas the vet, their concern is that they can’t get the owner to do what they think is needed.” Focus Group 5.*

This finding has implications for the origins of ethical conflict within teams and for team members’ understanding of one another’s roles, which is explored more fully in later sections.

With regard to relational barriers, participants described their frequent efforts to persuade other individuals of the right course of action and their ongoing uncertainty and regrets over eventual outcomes:

*“[I] went through her options a few times, for trying other medications, more pain relief, all this. The owner was becoming really upset at this point … so then it kind of came back to me … do I outright refuse? So we did go ahead with the put to sleep … but part of me does think, should I have fought back a bit more? But then where does that go? She doesn’t want this dog … Yeah, so it was a bit of a hard one for me.” Focus Group 2.*

The study also identifies examples of more specific relational obstacles to desired ethical outcomes, such as team members’ concerns for vulnerable animal owners:

*“We’ve had to withdraw pain relief from animals because we were suspicious that the owner is using them themselves. So again, we don’t want that animal to be in pain, but we do not want the client to overdose on meds we’ve supplied to the pet. I think it’s because of the social demographic that we serve, we see more cases like that. So we have a quite unique set of moral stress here.” Focus Group 5.*

In summary, the data analysis identifies and separates practical and relational barriers to ethical action, which are at play in the charity veterinary setting, with implications for how resulting moral stress is perceived and managed. Whilst practical barriers are more commonly accepted by team members, they often attempt to challenge relational barriers, potentially causing conflict within teams and with animal owners. Vets and nurses experience different practical and relational barriers, which are specific to their roles, and whilst some identified barriers may also apply in other veterinary settings, others appear specific to charity veterinary practice.

#### 3.1.3. The Impact of Veterinary Moral Stress

Participants were encouraged to explore what moral stress feels like and how it impacts veterinary team members. Moral stress was frequently described as causing feelings of intense frustration:

*“Sometimes it’s not knowing what the right thing to do is, but not usually. It’s usually kind of like a frustration, something you feel is appropriate morally and ethically, and you can’t do it.” Focus Group 4.*

Moral stress was also described as a feeling of powerlessness when team members accepted that there was nothing they could do to improve the ethical situation. Participants reported moral stress associated with an inability to act but also through being forced to take actions that they felt were unethical. Veterinary surgeon and veterinary nurse participants appeared to be differently affected by these specific situations:

*“When owners are not wanting to euthanize, we see them obviously as inpatients, [and] it can be really difficult to watch them suffer; like I can’t do anything about it, I just have to watch and wait. So, that’s definitely difficult. But as I say, I almost feel like there’s nothing I can do in that situation.” Focus Group 2.*

*“But I suppose, yeah, in some situations, you know that you’re doing maybe something that’s […] it’s the decision you’ve been backed into a corner of doing, and you feel like I’ve got no other choice.” Focus Group 7.*

As such, team members reported complex emotional responses associated with both failing to achieve their goals and also with undertaking practices that felt unethical. For example, team members related feelings of anger and failure associated with not achieving their ethical goals.

*“I think about this one occasionally as well, and how angry I was that it slipped through the net and it just got forgotten about. I feel responsible for that. Because I suppose part of it is because one of the things is my failure, and one of the things is the owner’s failure.” Focus Group 2.*

Furthermore, team members also reported feelings of guilt and regret associated with their role in achieving clinical outcomes which felt unethical:

*“So I think that kind of weighs heavy on your heart when you think, could I have pushed them anymore, could I have said something differently?” Interview 2.*

Team members reported feeling that there was sometimes no acceptable solution in the veterinary clinic and that choosing any outcome could feel like taking the “least worst option”:

*“The hardest cases are the ones in that there is an ethical mismatch or an ethical dilemma in them, and you have to try and do what’s right by everyone the best you can, even though you don’t really feel satisfied by any of the outcomes that are available to you.” Focus Group 9.*

With regard to the daily impact of moral stress, some participants reported “taking moral stress home”, potentially causing problems with relaxation, personal relationships, and sleeplessness:

*“They’re the cases that you think about at night, that you go home with, if you could have taken that pet, if you could have saved it and brought it home, you would have.” Focus Group 9.*

In some cases, participants explicitly described how their mental health had been affected by cumulative moral stress relating to working in veterinary practice:

*“Which, yeah, that sent me into a proper spiral of just horribleness […] I was a long time on antidepressant tablets because I got into … that rut of worrying about everything.” Interview 1.*

This study shows that moral stress has negative impacts on staff well-being, including on their lives outside of the clinic. Specific ethical challenges, as well as cumulative low-level moral stress, may be implicated.

### 3.2. Ethical Group Discussion

In this section, the practical challenges and benefits of ethical group discussion in veterinary practice are explored. The role of ethical group discussion and other approaches for reducing moral stress in veterinary teams is also described.

#### 3.2.1. Practical Considerations and Ethical Group Discussion

The participants did not have any previous experience of structured ethical discussion in any veterinary practice. However, participants felt that there was a good culture of openness and that colleagues were willing to discuss active cases together:

*“I think there is a healthy degree of openness, where colleagues feel they can touch base with other colleagues, where they feel that it’s not an easy decision if only to share the burden.” Focus Group 1.*

In this way, an existing ethical discussion was described as largely informal and related to supported clinical decision-making for active cases rather than ethical reflection:

*“I generally think that we do have quite a lot of conversations about the ethics of cases, even if it’s indirectly, we don’t always sit down and debate it, but I often, I find that I’m going out to prep, and … kind of being like what does everyone think? What would everyone think about this certain situation? And then you get everyone’s opinions that’s in the room, and it kind of helps you gauge and get a bit more of a footing as to what we’re gonna do.” Focus Group 2.*

In spite of this, participants reported that they would still appreciate more regular formal opportunities to review complicated cases as a team:

*“I think clinical notes are great, but sometimes it’s the combined, it’s like three vets and three nurses have seen a pet on six different days, and we’ve all drawn a slightly… You know, we’ve all taken something slightly differently from that, that it’s not pulled together to be as cohesive as it maybe should be. So I suppose for a cohesive decision to be made, if there’s a particularly complicated case or it’s complicated by different factors, it would help reduce stress, it would help in the decision-making for the entire case, improve welfare of the pet and of the staff involved.” Focus Group 9.*

Whilst such formal team review of active cases was not reported to be usual practice, participants described how this improved patient care when it did occur:

*“We had one happen a couple of weeks ago sort of by accident actually, where probably, I think probably half the team had concerns about a specific dog and a case, but that we hadn’t really all voiced those concerns together all at the one time, and when we did the course of action for that dog completely changed. I think it is something that ideally would be factored in on a weekly basis.” Focus Group 9.*

In addition, team members described how their anxiety over the treatment of complex cases had also been alleviated through a group discussion of the case:

*“It had been preying on my mind for days … [my manager] was like, ‘right, let’s all go downstairs, it’s in the building now, we’ll all have a chat’. And there was about six of us that were involved in the chat at various levels, and it was really nice. I left thinking, ‘oh I wish I had just done that earlier’, but it was a real comfort to me.” Focus Group 2.*

What was not currently experienced by team members through existing arrangements, however, was an ethical reflection on previous experiences and cases:

*“I suppose just maybe reflective time because I think we do have quite a lot of staff meetings, but we don’t necessarily reflect on cases as a group. And sometimes there’s so many of us, and we all, it might take long to go through it all, but I think it would be quite nice in some situations just to have group… like a debrief on some things. Especially because we’ve never done that before with any ethical problems, we’ve always just kind of maybe had a bit of a discussion at the time and then never reflected again.” Focus Group 4.*

In this study, a distinction between the purposes and benefits of ethical review of active cases and ethical reflection became quite clear. Participants felt that ethical reflection specifically had the potential to improve staff relationships but also to improve animal patient care in the long term:

*“We had a few, like, quite heated discussions because people were really, like, cared for the animal and really wanted to do the right thing. And then afterwards… I don’t think there was any animosity between staff, but I think it would have been nice if we could have all chatted about it when the situation had been dealt with, so then we could all kind of hear each other’s opinion, maybe like a more relaxed environment where people would… There was kind of less emotions involved. And again, maybe in some situations here, we do have quite a lot of palliative care for a lot of animals … it would be useful to discuss that after some cases for the animals’ wellbeing as well.” Focus Group 1.*

This study suggests that time is a more significant limiting factor in developing reflective ethical discussion away from the clinical setting than it is for developing active case review, which could happen during routine daily interactions in the clinic:

*“I think in the nature of how we work, we don’t necessarily have that much time in for in-depth discussions in the moment. A lot of decisions do tend to be ad hoc, in that you’d think well I’m not quite comfortable as to which way I jump with this, I’m leaning this way, do you think I’m within my rights to think that way? And that’s kind of your discussion done, simply because that’s about as much time as you’ve got in that moment.” Focus Group 1.*

The data analysis indicated a need to reassure participants of the importance and value of reflective approaches in order to avoid worsening their feelings of guilt and stress. Some participants reported concern about time spent on the focus group discussions and whether this had impacted negatively on the rest of their team and their animal patients:

*“It’s made the people that are doing it think about it a lot more, and I don’t think it’s caused us particularly any more stress, but I do wonder, with all of us out of circulation, the impact for the rest of the team.” Focus Group 8.*

Thus, whilst further developing active case review was considered feasible, team members were clear that it would take considerable investment from their organization to enable reflective conversations to regularly take place:

*“I think it would work. Think it would just be getting management on board to give the staff time to actually have those conversations.” Focus Group 9.*

Participants could see the value of involving both veterinary surgeons and veterinary nurses in ethical group discussions as a way to improve communication and reduce ethical friction between veterinary and nursing teams. This connects with observations surrounding the need for vets and nurses to better understand one another’s thought processes and the different relational causes of moral stress that they experience:

*“I think as a vet team we can normally come to a consensus fairly easily. But I don’t know whether animosity’s the right word, […], but I think perhaps there is like one team discusses with each other and another team discusses with each other, and yeah, there’s less of an interaction between the two.” Focus Group 6.*

However, team members observed that there was no existing structure for ethical reflection as a group and that they would need support in setting up such an initiative:

*“I don’t think we know how to do it properly as a group. If I was honest, I don’t think we know how to talk to each other properly as a group and to manage these things properly as a group.” Focus Group 4.*

According to this study, the major challenges for embedding ethical reflection within veterinary teams are the time commitment that would be required and how constructive discussion could be facilitated:

*“Although we have meetings every single week, they’re usually only about half an hour, more often than not 20 min into them, the receptionist comes and says there’s an RTA, or there’s somebody lame, or somebody’s rocked up without an appointment, and you get dragged away from them anyway. So I think if it was well structured, with enough time given to groups of staff to be able to have those conversations, and also be taken seriously because I can imagine if you did put loads of people in a room and say chat amongst yourselves, they would probably talk about telly!” Focus Group 9.*

In summary, team members value the informal conversations they have about the ethics of active cases in the clinic, although this could happen more frequently for the benefit of patients and staff. They would also like to be able to reflect on the ethics of previous cases as a mixed group of vets and nurses but would need organizational support to find the time and appropriate structure for such an approach.

#### 3.2.2. The Value of Ethical Reflection

During this project, the focus group discussions became a model of facilitated ethical reflection within small groups of colleagues who work together in the hospital setting. The participants were encouraged to explore the benefits and drawbacks of their experience. Feedback from participants was largely positive, and aside from the time commitment, team members did not feel that the experience had significant negative aspects, although the challenge of listening to what some colleagues wanted to say was raised:

*“I don’t think it’s created more stress, and it’s certainly been very interesting. And yeah, some of the comments that some staff have come out with have surprised me a little bit. But yeah, as [Vet] says, it would be really nice to be able to discuss this in depth more often, with all or any staff, but it is a time factor, really.” Focus Group 8.*

Even close team members described how they had not otherwise heard their colleagues’ individual points of view:

*“I know all those others in the group quite well, like even personally, but I heard lots of things I would never have thought that they would think.” Interview 4.*

*“It’s one of those, like, you just, you work with these people every single day, but until you actually sit down and ask those questions, you just don’t really know how their, how their heads are working or what’s going on or anything like that.” Interview 1*

One benefit of group ethical reflection was, therefore, exposure to the viewpoints and perspectives of other team members:

*“It was really helpful, it was really interesting to hear, like, different people’s opinions. Because you don’t really get, like, that kind of discussion otherwise unless you have focused time for it.” Interview 4*

In particular, participants felt this was a valuable opportunity to discover how others felt about specific issues:

*“That’s the bit I liked about it the most probably, the bit when [vet] was mentioning the dog that she was worried about, and actually until she brought it up to the team leader and made a point of saying, I’m really worried about this dog’s… Like, the care we’re giving this dog. And then you noticed actually there was four other people in the building that thought exactly the same … I just really enjoyed it.” Interview 1*

Vet and nurse team members described how the group discussion had made them more aware of each other’s roles and increased their understanding of why they might not always view things in the same way.

*“But I’ve enjoyed getting the insight into how the vets work out their process, and [Vet] said to me last week when we were having a chat about it that she had never really given a thought to the fact that nurses don’t [always] know what happens in a consult […]. And so she was, like, it’s just really strange getting it from your point of view because I just would always have thought that you knew what happened there, like, that was just always my assumption. So yeah, no, I totally loved getting the insight into how the vets think, how the vets work, and their kind of ideology on what it means to them to be a vet at [Organization], and how it differs between some of them as well.” Focus Group 6.*

Nurse team members, in particular, found the process helpful for understanding the relational complexities of the veterinary consult and the reasons why decision-making with animal owners can be especially difficult:

*“But actually, you forget then that that vet has to go back through and convince an owner who’s loved that pet for, like, 14 years, to put their animal to sleep, and you forget the pressure that puts on the vet. So that opened my eyes to that.” Interview 1.*

A prominent feature of the group discussions was the potential for reflection to inform future decision-making and to alleviate individual concerns about previous clinical decisions:

*“I suppose if there’s a future similar case, that you can go back to see what decisions were made in the past, how did we approach it, how would we approach it differently the next time? I think maybe to give someone a bit more clearance, you know, instead of going home and thinking about it, oh no, did I do the right thing? Being able to bounce it off someone else and say, ‘Yeah, I can see why you made these decisions, I think at this time you did the right thing, I can see your reasoning behind it’, just give you that peace of mind as well.” Focus Group 9.*

There were many examples during the discussions of team members actively supporting one another through their reflections on difficult decisions:

*Participant 1: “Maybe morally, I felt that was the wrong thing to do, but kind of situationally, it was the right thing to do. Sometimes you do feel quite conflicted.”*

*Participant 2: “I think if you hadn’t, you’d have left that dog at risk of undue suffering.”*

*Participant 3: “Yeah, it was the right thing to do.”*

*Participant 2: “It wasn’t the time and the place you wanted to do it, but it was the right thing. And your ability to choose the time was taken away from you by the owner’s circumstance.” Focus Group 4.*

Team members described how the ethical discussions helped to alleviate feelings of guilt and provided reassurance:

*“You can understand where … other people are coming from, or perhaps you can mitigate some of the misgivings that you have about something because they’ve explained how they can justify it to themselves.” Interview 4.*

*“And also, I think then if someone else has had a similar situation, it makes me feel a bit, maybe, less guilty or, like, some sort of reassurance that someone else will have done a similar thing.” Focus Group 4.*

The group discussions also provided a space for team members to explore some of the operational reasons why ethical problems might arise in the clinic and how the situation could be improved:

*“I think sometimes the problem is vets don’t want to step on each other’s toes and interfere in each other’s cases. Which I understand, and very often, people, maybe nurses, maybe VCAs, are not happy with what’s happening but won’t speak up, and the people that do speak up can feel quite unsupported.” Focus Group 5.*

Participants felt that their improved understanding of each other’s roles and perspectives might help them to discuss areas of conflict more constructively in the future:

*“And so I think that I came away thinking like OK, if there is a situation where I feel like people are a little bit judgmental of what the vet’s doing, kind of explaining to them what other kind of factors are coming into our decision-making might be enough to kind of bring them around, create less… Not conflict, but you know, resolve feelings.” Interview 2.*

Overall, the participants felt it was an experience they would like to repeat more regularly, with some offering ideas to develop such an agenda:

*“But certainly, having like two or three vets, different days of the week, different times of the week, because we’ve got lots of part-time people who can never make, like, the same meetings, but mixing it roundabout so that everybody gets a little bit of a turn to get an idea about what the meeting was about and what kind of went on, and get to air their views. No, and [Vet] was up for it, she said basically if I can get something, like, down on paper, about how it would work and how we would block the diaries down to accommodate it and things like that, she was totally up for starting it and getting it, getting the ball rolling with it as soon as we could.” Interview 1*

In summary, ethical group reflection during the focus group discussion was a positive experience for team members, although it could be surprising and difficult to hear some of their colleagues’ viewpoints. Participants appreciated hearing the different perspectives of other team members and understanding how ethical decision-making within vet and nurse roles differed. Ethical reflection was helpful for alleviating feelings of guilt, providing reassurance, and informing future decision-making. Participants actively supported one another during the group discussion and felt more able to have constructive conversations over potential areas of tension in the future. Participants also shared new ideas and advice during the discussions and explored areas for improvement in communication within and across teams. There was considerable interest in developing more permanent opportunities for ethical reflection within hospital teams.

#### 3.2.3. Reducing Moral Stress in Veterinary Teams

Whilst the ethical group discussion did have many positive benefits, the identification of practical barriers to ethical action as a cause of moral stress highlighted some limitations of narrative approaches to addressing the problem. Identifying and acknowledging practical barriers was helpful for participants; however, they were keen to point out that where a practical barrier existed that they believed should be removed, repeated discussion could be counterproductive unless a practical effort was made to reduce the impact of that barrier:

*“I think it’s just like having the discussion and then having an outcome as well. I think a lot of discussions happen, like there and then, and it’s always no outcome, […] think that’s a frustrating thing, yeah, I would say. There has to be an outcome at some end … It’s always the same thing as well, over and over again, the same topics over again, people say the same things over again, and nothing happens.” Focus Group 1.*

This has implications for veterinary practices in that ethical discussion may result in the identification of practical barriers which are within their power to control. In this case, practices and organizations must take responsibility for reducing the impact of such barriers if they wish to reduce the effects of moral stress on their teams.

For moral stress caused by relational barriers, ethical group discussion appeared particularly helpful since it enabled team members to develop a better understanding of one another’s perspectives and roles. As such, many participants reported that they felt better equipped to understand why other individuals may not make the same decisions as them. This had the effect of reducing the impact of perceived relational barriers by altering individuals’ opinions on what an ethical course of action might be:

*Participant 1: “I would say 90% of the time, the nurses probably clock that at 10 past nine in the morning when you’ve admitted it, and know that that’s gonna be the outcome at the end of the day [euthanasia]. Sometimes it takes [vets] a bit longer to persuade clients round to that way of thinking, or to just be sure in your head that there’s not another option available.”*

*Participant 2: “I think possibly after our discussion last week when I went away and thought about it … I never really considered the fact that the vet would have to go away and … convince somebody that doesn’t want to give up their wee pet that actually that is the best case. So it made me rethink, maybe I’m too quick to say, ‘Put to sleep’. And I didn’t say it today!” Focus Group 3.*

This finding demonstrates that during the lifetime of the study, the ethical group discussions resulted in a positive change in group dynamics and reported behavior change within the team. In this example, the nurse reviewed when they might suggest euthanasia to the vet because they better understood the reasons why this course of action might not always be immediately possible. With regards to relational barriers arising through disagreements with the animals’ owners, the ethical discussion groups are less likely to be effective at reducing moral stress since the owners themselves are not involved. However, there were also examples of team members becoming more accepting of the owner’s likely viewpoints after reflecting on specific cases with their colleagues.

During the course of the project, participants offered other potential approaches to dealing with moral stress. Quite commonly, team members suggested that they tried to distance themselves from emotional engagement with the animal and allow others to take responsibility for the decisions made:

*“So, yeah, I think that’s probably, it was a difficult, like, constant brain training of ‘it’s not your pet, you just need to give them the information, and it’s up to them to do with it what they will’, yeah.” Interview 1.*

*“As horrible as it would be for the animal because it’s not gonna be a nice passing for them, but in my mind kind of ‘well it’s up to the owner, so it is their decision’. And I’m kind of generally quite chilled about things … Kind of it irritates me that they do it, but at the same time, I just kind of accept it.” Interview 3.*

Some team members suggested that they tried to avoid contact with suffering animals when they knew they would not be able to improve the situation, although they did not suggest that this was an acceptable solution:

*“That’s when the moral stress comes in. When you’re looking at an animal suffering, that’s when it’s gonna be hard, isn’t it? I consciously don’t go look at the animals because I know it would upset me… I would purposefully not go out there to see it. So it’s not the answer at all, that’s just where I’ve got to.” Focus Group 5.*

Veterinary team members also recognized the potential role of their own personalities in creating the feelings associated with moral stress:

*“And, of course, we’re all perfectionists, aren’t we? We want to do everything right and get the right decision made, the right thing done at the end, so if we can’t do that … yeah.” Focus Group 2*

As such, some members described the need to be able to forgive themselves for not achieving the ideal outcome and for accepting the part that others play in ethical decision-making:

*“And I think, you know, it’s coming to that realization that you couldn’t have changed it, they have a very steadfast mindset, you’ve done all you can for that animal.” Interview 2.*

When this proved to be difficult, participants benefitted from the informal sharing of their concerns with colleagues:

*“You’re left with a feeling that you’ve failed your patient for reasons out of your control, but you kind of feel it’s your patient, you should be in control. So, sometimes it’s kind of hard to cut yourself some slack for doing your best in a difficult situation. So I kind of minimize my own personal moral stress by things that feel iffy, I make sure I share it with someone. You know, like, this is how I see it, would you do the same? And then if they say no, then maybe it gives me the backbone to go ‘ [I’m]not having it, we’re doing [something] different’, or if they agree, then at least it’s not just me that thinks that. So sharing helps.” Focus Group 1.*

Not taking moral stress home appeared easier for some team members than others, and participants reported using exercise, reading, and audiobooks to help distract them:

*“But basically, when I leave the building at the end of the night, work’s gone. […] I’m not thinking about work anymore. And that’s something that a lot of people don’t… Like they really struggle with shutting off work.” Interview 1.*

In summary, where practical barriers to ethical action exist, they may need to be addressed as well as discussed to result in the reduction of moral stress. Ethical group discussion appeared potentially effective for reducing moral stress caused by relational barriers within teams. Exposure to the realities of one another’s roles and thought processes led to an improved understanding and acceptance of different ethical perspectives. Ethical group discussion resulted in improved group dynamics and positive behavior change within the life of the project, and participants were generally keen to develop a longer-term approach. The main concerns raised were the required investment of time and the need for structure/facilitation. Team members’ alternative strategies for reducing moral stress included distancing themselves from animals and allowing others to take responsibility, distraction, switching off at the end of the day, forgiving themselves, and sharing concerns with colleagues.

## 4. Discussion

This study aimed to qualitatively evaluate the use of ethical group discussion for its potential to reduce moral stress in charity veterinary teams. The study responds to recently published work which highlights veterinary staff dissatisfaction with the level of ethical discussion in their workplace, alongside a hypothesis that open discussion centered on ethical reasoning, ethical dilemmas, and reflection on cases may be useful as a possible management tool for reducing conflict and moral stress [18]. The results of the study confirm the desire to engage in ethical discussion more frequently within veterinary hospital teams. Specifically, whilst staff members reported frequent informal discussions of ethical aspects of active cases, they requested greater investment in facilitated ethical reflection. Veterinary organizations more broadly may wish to reflect on the need to invest in both these activities as an important but currently underdeveloped aspect of staff training and well-being for the veterinary profession [11].

This study develops our understanding of the concept of moral stress in veterinary teams. Whilst moral stress was not a familiar term for all participants, they found the phrase useful and intuitive in describing their everyday experiences. The veterinary literature has connected ethical dilemmas with differing degrees of workplace stress [8], and the most recent empirical work in this area identifies moral injury [14] and moral distress [13,17] within the veterinary profession. Interestingly, the terms “moral stress” [8,10], “moral distress” [13,17], and “moral injury” [14] appeared to be used interchangeably by some participants. Whilst specific ethical conflicts were discussed for their role in causing moral stress and distress [8,17], the participants also frequently reported a more insidious and everyday uncertainty surrounding their ability to fulfill their ethical obligations; thus, the data analysis highlights the existence of continuous low-level moral stress in charity veterinary practice, in addition to moral distress caused by more dramatic ethical challenges. According to the “sliding scale” understanding of moral challenges and corresponding responses [15], this study suggests that frequent, low-level moral challenges in the veterinary clinic can result in feelings of everyday stress for team members. The mental health consequences of moral stress, therefore, appear to overlap to some degree with those attributed to moral distress arising from particularly stressful cases [17] or moral injury as a response to imposed unethical practices [14]. Importantly, this study’s results also suggest that moral stress may be cumulative in nature and may also interact with other forms of workplace and personal stress. Future work may usefully focus on the boundaries and interrelationships between these three concepts (moral stress, distress, and injury), including their role in veterinary mental health.

The study supports previous work in the field, which has identified feelings of frustration, powerlessness, anger, guilt, and failure associated with moral distress [17]. An important finding from this in-depth study, which may help to explain these complex emotional responses, is that team members sometimes feel that no acceptable outcome is possible in the veterinary clinic but that they are compelled to act, nonetheless. Thus, team members do not only fail to achieve an ethical outcome, which causes frustration and anger, but they feel guilt and regret over the course of action they must take instead. This aspect of the study results confirms a need to properly identify and label moral conflict [13], for whilst these emotional responses appear familiar within veterinary teams, the concept of moral stress and the underlying reasons behind these feelings are not currently well-recognized. The study results also support recent work which proposes that complex emotional responses in the veterinary clinic may alert us to specific complexities in veterinary ethical decision-making and that emotional aspects of veterinary practice might become more centered within contemporary veterinary ethics approaches [32]. With regard to the well-being of veterinary team members, their frequent reporting of such emotional experiences at work underlines the mental health implications of moral stress and the urgent need for increased organizational and professional support.

This study adds depth to our understanding of the multiple obstacles to ethical activity at play in the veterinary clinic [11]. An important insight from this analysis is the identification and separation of practical and relational barriers and the differing reactions and responses to these obstacles reported by participants. Whereas practical barriers may often be accepted, even when they cause moral stress, relational barriers appear to be more regularly resisted through attempts to convince others to change their viewpoint. This finding is important since it highlights that different strategies for dealing with practical or relational obstacles and for coping with the resulting moral stress may be necessary. For example, the results show that repeated discussion of moral stress caused by practical barriers may be counterproductive, particularly when team members feel that these barriers could be effectively reduced through organizational or other interventions beyond discussion/dialogue/conversation. In contrast, group discussion did appear potentially helpful for alleviating moral stress caused by relational barriers through altering team members’ perspectives on what constitutes an ethical outcome.

This study also identifies the potential for different forms of moral stress to affect different team members; for example, vets and nurses identified different relational and practical obstacles to achieving their desired ethical outcomes. Therefore, whilst ethical discussion between vets and nurses appears important for reducing friction within teams, it may also be necessary to consider the specific causes of moral stress for different team members in the hospital setting. The identification of both practical and relational barriers which appear specific to charity veterinary work raises the likelihood that teams working for different veterinary organizations will also experience different practical and relational obstacles, dependent on the policies, relationships, aims, and expectations in each setting. Future work on moral stress will need to remain open to the complexities of both practical and relational barriers to ethical action, as described in this study, and to engage with the need for a bespoke understanding of each team’s challenges and requirements in their specific work setting.

This study demonstrates the speculative use of an alternative facilitatory approach to that reported by Quain et al. [17]. In this study, the facilitator did not make specific reference to ethical theory or alternative clinical outcomes [17]; instead, the participants were encouraged to use the VET [21] to explore cases through the lens of multiple veterinary relationships. In addition, participants were encouraged to reflect on the concept of moral stress, as defined by Reynolds et al. [10], and to explore their own and others’ emotional responses to ethical conflicts. The data analysis provides considerable detail on the benefits and challenges of using the VET as a tool for structuring ethical reflection as a group, which is planned for separate publication. It is important to note here that the tool’s identification of multiple ethical *relationships*, rather than multiple *stakeholders* [22], within the veterinary setting was useful in this study for separating practical and relational barriers to solving ethical challenges. The VET also helped groups to identify and discuss the specific relational barriers which exist within different veterinary roles.

It is interesting to note that the findings of Quain et al. [17] overlap with the findings of this study in many important ways. As such, it may be the case that the specific facilitation style and frameworks, which are used to structure the ethical discussion, are less important than the opportunity to discuss veterinary ethics as a group. The approach taken in this study is important in demonstrating what may be achieved *without* formal reference to ethical theory or specific clinical approaches, making space for the development of facilitated ethical group discussions which can engage staff at all levels using language which is already familiar to them. This approach may make it easier for staff to express themselves and their experiences and also opens up the possibility of ethical group discussion being facilitated internally by trained staff who may not have previous qualifications or experience in veterinary ethics [11]. Indeed, it may be the case that existing in-house facilitated forums for discussion which are used in other healthcare settings, e.g., Schwartz Rounds [33], could be reoriented toward group ethical discussions in the veterinary clinic. Schwartz Rounds are not explicitly connected with ethics in other healthcare settings but are focused on emotional and social aspects of healthcare practice; however, the more formalized ethics support which already exists in these settings [11] may explain how this distinction is possible. Alongside later assertions that emotional and social aspects of healthcare practice *are* ethical in nature [32], this study also considers that in the veterinary setting, where ethical decision-making is often still considered a personal and autonomous responsibility, implementing these types of discussion forums would be likely to represent a valuable opportunity for ethical reflection beyond the individual.

This study contributes new knowledge with regard to the benefits of distinguishing active case review from ethical reflection. Whilst previous work has highlighted the need to incorporate both dimensions within an ethics support service [11,17], in this study, they were proposed as ideally separate undertakings by the participants for both practical reasons and to allow ethical reflection to occur in a more relaxed manner. This study indicates that ethical reflection, when distanced from specific problematic events, may alleviate moral stress through an improved understanding of ethical complexity and alternative perspectives, even where clinical outcomes may not be changed. This study, thus, highlights the importance of separating the processes of reflective ethical group discussion and active case review in order to maximize positive outcomes. This finding is important because it shows that veterinary organizations will need to consider how both of these opportunities will be afforded to their staff.

The benefits of ethical group discussion in this study illustrate in detail those identified through survey methods by Quain et al. [17], including helping to clarify thinking, seeing ethical challenges from the perspectives of others, providing a safe and supportive environment, identifying and dealing with moral distress, validation of ethical decision making and increasing confidence to speak up in the workplace [17] (p. 11). Taken together, these studies thus provide valuable confirmation that ethical group discussion may be anticipated to have such benefits, irrespective of the specific facilitation arrangements and group compositions. The potential for negative emotional impacts from ethical discussion and the effect of power hierarchies in preventing open discussion of ethical challenges identified by Quain et al. [17] was also evident in this study’s findings. However, this study highlights that these concerns also exist in practice more widely and that whilst they may be inadvertently recreated within the discussion group situation, there is also the potential to ameliorate these problems to some degree through positive experiences of *regular* facilitated ethical discussion with a small and varied group of close colleagues. The longitudinal nature of this study is particularly relevant because the results demonstrate how the participants began to reflect together on their experiences during a previous session and the ways that it has altered their thinking about themselves, their decision-making processes, and their colleagues. This study shows that regular discussions within hospital teams have the potential to positively change team dynamics and alter behavior. Negative findings concerning ethical discussion, which arose from this study, included the challenge of listening to what some colleagues had to say, the repeated discussion of practical barriers to solving ethical dilemmas without identifying a solution, and the potential for further guilt and stress to be caused by taking time out from clinical duties.

The most prominent challenge to implementing effective ethical group discussion at an organizational level [11] was perceived to be a lack of time and a need for formalized organizational support and facilitation. The participants offered their own alternative suggestions for how moral stress might be managed; these included distancing themselves from animals and allowing others to take responsibility, distraction, switching off at the end of the day, forgiving themselves, and sharing concerns with colleagues (Richards et al. 2020) [34]. It is important to note that, in line with the reported benefits of ethical group discussion, these practices also appeared to assist veterinary team members in dealing with the realities of not being able to provide so-called “gold standard care” [14]. As such, the study connects with recent work which problematizes the “gold standard” concept and promotes the necessity of contextualized approaches to evaluating veterinary care [35]. In the context of the veterinary charity hospital, this study indicates that team members may benefit greatly from alternative measures of success in veterinary treatment, which more adequately reflect the realities faced in practice.

As with all research studies, this project does have some limitations which must be acknowledged. Firstly, the size of the study is relatively small, which means that further work would be needed to determine the extent to which the findings extend to other teams, hospitals, or veterinary settings. The study was also limited by practical considerations since it was difficult for hospital teams to commit to removing staff from the rota regularly to participate in the research. This meant that there was sometimes several weeks’ delay in conducting consecutive focus groups and that some participants were not available for all the focus group sessions. The collaborative design of the research meant that there was some compromise required in order to satisfy the requirements of the organization. The use of the VET [21] and its specific focus on COVID-19 work are examples of research dimensions that were proposed by the organization rather than introduced by the researcher. The use of the VET [21] to structure the ethical discussions resulted in the consideration and critique of one specific approach to veterinary ethical decision-making. Other approaches could also be explored, including the use of alternative tools, such as the Ethical Matrix [22]. An additional limitation of collaborative ethics research exists with respect to how much data it is appropriate to publish, as opposed to more private internal reporting. The study results, however, highlight the potential for successful co-produced ethics research to generate meaningful and impactful results in specific veterinary clinical settings and high-quality academic outputs for wider audiences.

The in-depth qualitative research described here represents a particular approach to ethics research that might be complemented or challenged by the use of other methods. Whilst some survey research already exists in this area [13,17], other qualitative methods, such as participant observation/ethnography [36], may reveal additional dimensions, specifically with regard to observable actions alongside narrative framings of moral stress and ethical decision-making. Furthermore, the qualitative evaluation of the ethical discussion presented here cannot definitively prove or quantify a reduction in veterinary moral stress. The research was initially planned to be conducted in person but became online due to continued restrictions on visitors to the hospitals following the COVID-19 pandemic. Whilst the participants themselves were able to converse within the same room wherever possible, the remote location of the researcher will inevitably have altered the research interaction to some degree. The dual role of the researcher in both facilitating ethical discussion and evaluating its value may be considered to have increased the participants’ likelihood of reporting only positive outcomes, for example, due to social desirability bias. In an attempt to mitigate this limitation, the researcher was clear about their interest in both positive and negative experiences, and participants did report limitations and challenges associated with the VET and their experiences of ethical group discussion.

Co-producing veterinary ethics research with a veterinary organization is unusual and was important in this study due to the considerable commitment required for a longitudinal approach to exploring moral stress and for developing potential management strategies within hospital teams. Furthermore, the novel use of focus group discussions, both as a research data source and a trial intervention, has led to both unique research data and organizational support for the further development of facilitated ethical group discussion. This collaborative aspect of the study design should be contemplated in terms of its role in shaping the developing field of empirical veterinary ethics towards research which is focused on practical change and which has the potential to generate impact in the short term. Whilst this research was conducted at a named organization, its findings are very likely to have an impact across the profession more widely.

Finally, the study also demonstrates the use of novel methodological and analytic approaches in veterinary ethics research. Whilst the use of in-depth qualitative methods in veterinary research more broadly is a relatively recent development [37], this study shows the value of such data in adding depth and detail to our understanding of key ethical concepts, such as moral stress and its significance within the lived experiences of veterinary team members. The semi-structured approach to data collection employed in this study left space for participants to determine the direction of their conversations and led to important and unexpected findings which may not have been captured using more prescriptive approaches. Overall, the semi-structured, co-produced research approach applied in this study, its creative use of facilitation, which was designed to allow participants to contribute using their own voice, and the structural and analytic attention paid to relationships and emotions in the veterinary clinic may permit this research to be described as a contemporary feminist [32] rather than traditional bioethical empirical study. This type of research approach may prove important as a further development in the application of “bottom–up” rather than “top–down” approaches to veterinary ethics research [11], where the detailed experiences of those working in the field may be thought of as critical in advancing our understanding of ethical veterinary practice, rather than veterinary ethics being viewed as a form of knowledge which must be imposed on the profession.

## 5. Conclusions

This study identifies moral stress as a recognizable and commonly encountered problem in veterinary teams. Moral stress may be caused by a continuous or frequent inability to achieve desired ethical outcomes in the clinic. Moral stress may be cumulative and can also interact with other forms of workplace or personal stress. Moral stress impacts negatively upon staff well-being, causing feelings of frustration, powerlessness, guilt, and failure, and can contribute to poor mental health. Obstacles to ethical activity may be either practical or relational in nature, and different team members may experience different barriers within their roles. Facilitated ethical group discussion may reduce moral stress caused by relational barriers by helping team members to better understand one another’s roles and ethical decision-making processes. Regular ethical discussions within veterinary teams have the potential to positively change team dynamics and alter behavior. Moral stress caused by practical barriers may also be usefully explored during the ethical discussion, but practical solutions aimed at reducing the impact of these barriers should also be considered where possible.

## Data Availability

As a consequence of research co-design, the data from this study cannot be made publicly accessible as sensitive commercial and contextual information relating to the named organization, their staff, and clients may not all be removed whilst retaining the data’s usefulness.
